# Advances in the prevention and treatment of postoperative delirium by acupuncture: A review

**DOI:** 10.1097/MD.0000000000033473

**Published:** 2022-04-07

**Authors:** Jian-Bing Yang, Long-Fei Wang, Yun-Fei Cao

**Affiliations:** a School of Medicine, Ningbo University, Ningbo, China; b Department of Anesthesiology, Beilun District People’s Hospital of Ningbo, Ningbo, China.

**Keywords:** acupuncture, postoperative delirium, prevention, treatment

## Abstract

Postoperative delirium is a common postoperative complication of neurocognitive dysfunction, especially in elderly surgical patients. Postoperative delirium not only damages patients’ recovery but also increases social costs. Therefore, its prevention and treatment has essential clinical and social significance. However, due to its intricate pathogenesis and limited pharmacological interventions, the effective prevention and treatment of postoperative delirium remains a thorny problem. As traditional acupuncture therapy has been shown to be an effective treatment in many neurological disorders, and in recent years, it has begun to be clinically used as an intervention for postoperative delirium. Although most clinical and animal studies confirm that multiple types of acupuncture interventions can alleviate or prevent postoperative delirium by relieving acute postoperative pain, reducing the consumption of anesthetics and analgesics, attenuating neuroinflammation and neuronal lesions, while more evidence-based medical evidence and clinical validation are needed for these encouraging effects.

## 1. Introduction

Postoperative delirium (POD) is a neurocognitive disorder occurred most often in the hospital up to 1 week post operation or until discharge, which is characterized by short-term fluctuations in mental status, attention, and level of consciousness.^[1,2]^ The prevalence of POD varies from 5% to 50%, depending on the patient's age, type of surgery, and various medical settings. Among different age groups, elderly patients undergoing surgery are at the highest risk of developing POD.^[3,4]^ As a crucial postoperative complication, a wide range of adverse outcomes have been demonstrated to be associated with POD, including cognitive dysfunction, prolonged hospitalizations, increased mortality and higher medical costs.^[5]^ Despite its prevalence and severity, to date, however, there are limited pharmacological interventions, and the exact pathophysiology is not yet fully understood.^[2,5]^ As an alternative and supplementary strategy, traditional acupuncture therapy has been tried for the prevention and treatment of POD with encouraging results, though more evidence-based medical evidence and clinical validation are needed. Herein, we review the clinical evidence and animal studies on POD prevention and treatment by acupuncture, and discuss the potential mechanisms involved.

## 2. Mechanism and intervention of POD

Despite the lack of a definitive mechanism for POD, growing evidence suggests that there are numerous underlying pathophysiological processes associated with POD, which is not a single entity. Advanced age, perioperative pain, neuroinflammation, oxidative stress, circadian rhythm or melatonin dysregulation, and even gut microbiota dysregulation have been shown to play crucial roles in the development of POD by interacting with each other.^[6,7]^ Some studies have also been focused on identifying the risk factors for POD, including elder age, multiple comorbidities, preoperative cognitive impairment, poor vision or hearing, and presence of infection.^[8,9]^ And due to these multifaceted causes and obscure pathogenesis, which remains poorly understood, it is obviously a challenge to prevent and treat POD. To date, there are few ideal interventions available for POD.^[10]^ Pharmacological interventions, such as haloperidolare, dexmedetomidine, nimodipine, with the aim of alleviating the incidence of POD, are frequently used in clinical POD treatment, and results from efficacy trials have shown early promise. However, their considerable side effects, including long QT syndrome, movement disorders, bradycardia, and hypotension, have greatly limited their clinical application.^[10–12]^ So many national or regional guidelines and expert consensus prefer non-pharmaceutic techniques to prevent or decrease POD, while drug therapies are only suitable for patients with hyperactive delirium.^[1,4,13]^ Meanwhile, nonpharmacological interventions for POD, with limited evidence so far, are showing advantages, among which acupuncture has been reported as a promising alternative to drug therapies.^[14,15]^ In comparison to pharmacological interventions for POD, long-term clinical application of acupuncture as an alternative therapy has shown that it does not have adverse effects on dependence, addiction, tolerance, or neurotoxicity, nor does it increase the metabolic burden of liver and kidney.^[13,16,17]^

## 3. Beneficial effects of acupuncture on POD

Acupuncture has been used for centuries in several East Asian countries, such as Korea, Japan, and China,^[18]^ to treat a variety of health conditions, such as gastrointestinal discomfort, chronic pain, and musculoskeletal disorders. Acupuncture uses fine needles or electrode sheets to stimulate specific physiological points, such as acupuncture points, myofascial points, or tender points, to regulate neuronal activity, as well as hormone and immune levels. At present, there are many types of acupuncture, including manual acupuncture (MA), electroacupuncture (EA), transcutaneous electrical acupoint stimulation (TEAS), transcutaneous electrical nerve stimulation, pharmaco-acupuncture, auricular acupuncture, acupressure, and dry needling, etc. Among them, the MA and EA techniques are the most common in clinical application, their related research and reports are also the most.^[19]^ Recently, acupuncture has also been used clinically to treat neurological or psychiatric disorders, including insomnia, anxiety, depression, schizophrenia, dementia, cognitive impairment, and delirium.^[20,21]^ A systematic review and meta-analysis confirmed that acupuncture can decrease the State-Trait Anxiety Inventory and Visual Analogue Scale scores of preoperative anxiety in patients, as compared to control groups or sham therapy.^[15,22]^ Acupuncture can also improve the cognitive function of dementia patients and reduce their agitation, a randomized study by Levy et al^[23]^ proved that acupuncture was safe and effective in the treatment of delirium, when served as an addition to standard-of-care, acupuncture might improve clinical outcomes related to delirium in older patients hospitalized in internal medicine departments, which was manifested in shortening time-to-first delirium remission, increasing the number of delirium-free days, and lowering the severity of delirium. Similarly, a combination of acupuncture and herbal medicine adopted by Matsumoto-Miyazaki et al^[24]^ was found to be effective in reducing the occurrence of delirium and exhibiting no serious adverse events in patients with cardiovascular disease in intensive care units. Another study has shown that, in patients with prior agitation, auricular acupuncture at tranquilizing points “Shenmen” and “Point Zero,” have a potential to manage postoperative problematic behavior such as aggression and agitation.^[25]^ Given the similar pathophysiological changes in POD characterized by neurocognitive disorders, it is not difficult to assume from these findings that acupuncture may also have beneficial effects on POD.

In fact, the benefits of acupuncture for POD have already been investigated in some clinical trials.^[19]^ Ding et al^[26]^ found that TEAS applied at the Shenting, Baihui, bilateral Neiguan, and Hegu points could reduce the dosage of propofol required during surgery and the occurrence of delirium after surgery in elderly patients, and the effect of preconditioning with TEAS before surgery was better than that with intraoperative application. A trend of reduced incidence of POD (6.3% vs 25%) was reported when geriatric patients with silent lacunar infarction received TEAS as an intervention before and during spinal surgery.^[27]^ Likewise, an accordant effect of acupuncture was observed by Fan et al^[28]^ at preventing delirium, which showed that the combination of TEAS and auricular acupressure reduced the incidence and severity of POD in elderly patients undergoing major abdominal surgery, as compared with standard care. Li et al^[29]^ found that scalp acupuncture performed during operation could reduce the incidence of POD and shorten the post-operative hospital stay in elderly patients undergoing hip replacement. The application of various acupuncture techniques, including EA, auricular acupressure, scalp acupuncture, etc., whether used alone or in combination with other interventions pre-, intra-, and postoperatively, have shown varying degrees of delirium-sparing effects, and with no significant side effects. These studies further expand the scope of clinical application of acupuncture, especially in the perioperative application, and are expected to show great clinical implications when serving as an adjuvant in postoperative delirium-sparing.

## 4. Potential mechanisms of acupuncture against POD

Although the mechanisms of acupuncture against POD are not fully understood, the most accepted one is pain reduction by acupuncture, as perioperative pain is considered to be the important predisposing factor for POD.^[6,7]^ Acupuncture, as a low-risk analgesic technique that has long been utilized in traditional Eastern medicine, has actually been incorporated into integrated clinical pain management in many countries around the world, including perioperative analgesia in surgical patients.^[30]^ A recent review has shown that acupuncture enhances perioperative analgesia, reduces the need for opioids, shortens time to return of bowel function, and decreases perioperative adverse reactions, such as nausea/vomiting. Patients undergoing abdominal, spinal/neurological, and gynecologic pelvic surgery generally benefit from acupuncture, and various acupuncture techniques, including MA, EA, TEAS, are promising as adjuncts to multimodal perioperative analgesia.^[31]^ As the main drugs for perioperative analgesia at present, opioids are also associated with increased risk of POD, opioid-sparing pain management strategies or analgesia alternative to opioids have become an important means to prevent and control POD.^[32,33]^ Among patients who received intraoperative application of opioids, there indicates an association with significantly higher odds of delirium and sleep disorders, which may impair cognitive function and explain the increased risk for POD.^[34]^ Other reports also confirm that acupuncture can alleviate acute postsurgical pain, reduce the demands for narcotics, and improve sleep disorders.^[35,36]^ Whereas a systematic review conducted by Wu and colleagues indicated that patients who received acupuncture had less postoperative pain 1 day after surgery, but it did not diminish the consumption of opioids, which was not entirely consistent with other reports.^[37]^ More studies have further revealed the best-known neural mechanisms of acupuncture analgesia, which may be related to adenosine A1 receptor,^[38]^ gate control theory,^[39]^ descending pathway,^[40]^ and brain areas mediated the putative analgesic effects.^[41]^ These clinical observations and mechanism studies have basically confirmed the analgesic effect of acupuncture and its important neural mechanism in POD prevention and treatment.

As POD has been hypothesized to be a precursor of postoperative cognitive dysfunction, the mechanism of acupuncture against POD may be largely similar to its effect on postoperative cognitive dysfunction, which is by attenuating systemic inflammation and neuroinflammation, reducing oxidative stress levels and neuronal injury, improving synaptic plasticity.^[42]^ A review also suggested that acupuncture, in addition to improving neurocognitive disorders through the above-mentioned mechanisms, is also associated with the reduction of anesthetic agent usage and the promotion of patients’ recovery.^[24]^ Other studies have also confirmed the anti-inflammatory and neuroprotective effects of acupuncture. Gao et al^[27]^ demonstrated that TEAS at bilateral PC6(Neiguan) and LI4(Hegu) points could alleviate POD in older patients with silent lacunar infarction, it's mechanism was related to reduce the neuroinflammation by lowering the permeability of blood-brain barrier. Similar findings were found in the study of Ding et al^[26]^ that TEAS at Shenting, Baihui, bilateral Neiguan, and Hegu points, could reduce the occurrence of postoperative delirium in elderly patients, the main mechanism may be related to the inhibition of inflammation and the alleviation of brain injury by reducing the plasma concentrations of neuron-specific enolization (NSE), tumor necrosis factor-α (TNF-α), and interleukin-1β (IL-1β). Similarly, Li et al^[29]^ found that intraoperative scalp acupuncture could effectively reduce the occurrence of POD in elderly patients undergoing hip replacement and shorten the length of postoperative hospital stay, the mechanism was also associated with ameliorating central nervous system lesion, and achieved through the reduction of inflammatory mediators and inhibition of inflammatory response. Another acupuncture technique, auricular acupressure, has been reported to exert its neuroprotective and anti-delirium effects by stimulating the auricular branches of the vagus nerve and activating the noradrenergic system of locus locus, one of the central relay centers of the vagus nerve, which plays a key role in the generating and regulation of delirium.^[43,44]^ By directly or indirectly regulating the activity and connectivity of the norepinephrine system in locus locus, auricular acupressure regulates the release and uptake of norepinephrine and dopamine in some key brain regions, including the prefrontal cortex and hippocampus, which are postulated to be associated with attention, memory, and other cognitive dysfunction.^[45]^ A review article by Hou et al^[46]^ is consistent with the above views, showing that auricular acupressure can relieve pain, treat insomnia and anxiety, and improve sleep quality, and its mechanism may be achieved by regulating the neuroendocrine system, neuroinflammation and neural reflex, as well as antioxidant effects. However, the detailed mechanisms of auricular acupressure against POD warranted further study.

Animal studies have also confirmed that acupuncture can reduce cognitive impairment and the associated pathological changes after various types of surgery. Yang et al^[47]^ found that EA stimulation at specific acupoints (e.g., DU20 + KI1) attenuated both surgical pain and delirium-like behavior in mice by a potentially important gut-brain interactive mechanism. Further studies showed that EA stimulation at specific acupoints could prevent neuroinflammation by reducing the activation of microglia at the spinal cord, somatosensory cortex, and hippocampus, and also affect the vagal-adrenal axis in mice.^[46,48,49]^ These findings revealed the new effects of EA stimulation on ameliorating pain, neuroinflammation, dendritic spine remodeling, and delirium-like behavior in animal models. Together with clinical findings, animal studies can provide insights for the future direction of research on the postoperative delirium-sparing effect and its mechanism of acupuncture.

## 5. Limits and major problems

Based on the studies included in this review, it seems possible to conclude that acupuncture has the potential to serve as a beneficial intervention and complementary therapy for POD. However, data quality of the current clinical studies is generally not high, and most of them have methodological problems and limitations, which may lead to potential bias. First, acupoint specificity has been controversial in acupuncture research. A number of clinical studies have found that acupuncture stimulation at acupoints based on traditional acupuncture theory have similar effects to the sham acupuncture points (including nonspecific acupoints or non-acupuncture points). That is to say, even sham treatment may produce benefits or curative effects. Therefore, the location of acupoints may not be as important as the stimulation techniques used in acupuncture treatments. Second, as acupuncture and related techniques are traditional practices in East Asia cultures and have high public acceptance, most of the acupuncture studies published so far have been completed in these areas, and it is possible to obtain higher satisfactory results than in other regions and ethic population without East Asian cultural background. So multi-center studies with different populations and regions are needed to correct this bias. Third, small sample sizes, unclear randomization procedures, inadequate blinding method, and the heterogeneity of study protocols are other limitations and problems. Objectively speaking, the overall methodological quality of these included studies is low, which significantly reduces the reliability of the data, the validity of the analysis results, and the repeatability of the studies. In addition, manipulation of acupuncture or stimulation parameters, including manipulation, frequency, intensity, and duration, may result in high heterogeneity and influence study results. Future studies need to remedy these deficiencies, and standardized methods and procedures should be emphasized in acupuncture studies.

## 6. Conclusion and prospect

The efficacy of acupuncture against POD is encouraging, which will present acupuncture as a recommendation for POD intervention to clinicians, health care policy makers, and patients. Nevertheless, more clinical trails with better study designs as well as high quality evidence-based proofs are required to confirm its effectiveness and the underlying mechanisms. With the trend of global aging, POD, being a common postoperative complication in elderly surgical patients, will attract increasing attention from researchers.

## Acknowledgments

This work was supported by the Traditional Chinese Medicine Science and Technology Project of Zhejiang Province, China (grant no. 2019ZA120).

## Author contributions

**Conceptualization:** Yun-Fei Cao.

**Project administration:** Yun-Fei Cao.

**Supervision:** Yun-Fei Cao.

**Writing – original draft:** Jian-Bing Yang, Long-Fei Wang.

**Writing – review & editing:** Long-Fei Wang, Yun-Fei Cao.
